
JOURNAL INFORMATION
==============================
NLM Title Abbreviation: J Am Heart Assoc
Journal ID: J Am Heart Assoc
NLM Journal ID: 101580524
Journal ID: jah3

Journal of the American Heart Association: Cardiovascular and Cerebrovascular Disease

EISSN: 2047-9980
Publisher: Wiley

ARTICLE INFORMATION
==============================
PMCID: PMC3540683
DOI: 10.1161/JAHA.112.000138
Article ID: jah3138
Article version: 1
Subjects: Editorial Board

Editorial Board

Publication date: 2012 Dec
Electronic publication date: 2012 Dec 19
Volume: 1
Issue: 6
Electronic Location ID: e000138
Copyright: © 2012 The Authors. Published on behalf of the American Heart Association, Inc., by Wiley-Blackwell.
Copyright year: 2012
License: This is an Open Access article under the terms of the Creative Commons Attribution Noncommercial License, which permits use, distribution, and reproduction in any medium, provided the original work is properly cited and is not used for commercial purposes.
License URL: https://creativecommons.org/licenses/by/2.5/

==============================
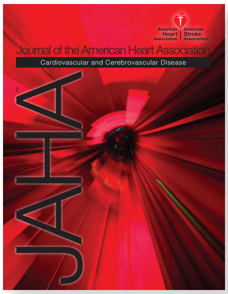

### JAHA: Journal of the American Heart Association
### EDITORIAL BOARD

### Editor-in-Chief
Joseph A. Vita

### Deputy Editor
John F. Keaney, Jr.

### Senior Associate Editors
Barry London
L. Kristin Newby
Thomas J. Wang

### Statistical Editor
Lisa M. Sullivan

### Statistical Consultants
Kevin J. Anstrom
Emilia Bagiella
Alexa Beiser
Serkalem Demissie
Kimberly Dukes
Michael LaValley
Qi Long
Janice Weinberg

### Editorial Board
Ivor Benjamin
Robert M. Bryan, Jr.
Holli Devon
Thomas Gerber
Mary Fran Hazinski
John Ikonomidis
Julie Johnson
Christopher M. Kramer
Pamela Peterson
David Pollock
Kerry-Anne Rye
U. Joseph Schoepf
Joseph I. Shapiro
Eric E. Smith
Jeffrey Towbin
Viola Vaccarino
Peter Wilson

### Managing Editor
Kathleen Sullivan

### Scientific Publishing Committee
Martha K. Cathcart, Chair
Richard W. Newman, Vice Chair

Hossein Ardehali
Salvador Cruz-Flores
John S. Floras
Kathy Griendling
Kelly A. Hadsell
Joseph A. Hill
Evangelos D. Michelakis
Beth Ann Rosner
Alvin L. Royse
Sean I. Savitz
P.K. Shah
Y. Joseph Woo
